# Inter-Slice Blood Flow and Magnetization Transfer Effects as A New Simultaneous Imaging Strategy

**DOI:** 10.1371/journal.pone.0140560

**Published:** 2015-10-14

**Authors:** Paul Kyu Han, Jeffrey W. Barker, Ki Hwan Kim, Seung Hong Choi, Kyongtae Ty Bae, Sung-Hong Park

**Affiliations:** 1 Magnetic Resonance Imaging Laboratory, Department of Bio and Brain Engineering, Korea Advanced Institute of Science and Technology, Daejeon, South Korea; 2 Department of Radiology, University of Pittsburgh, Pittsburgh, PA, United States of America; 3 Department of Bioengineering, University of Pittsburgh, Pittsburgh, PA, United States of America; 4 Graduate School of Medical Science and Engineering, Korea Advanced Institute of Science and Technology, Daejeon, South Korea; 5 Department of Radiology, Seoul National University College of Medicine, Seoul, South Korea; Brighton and Sussex Medical School, UNITED KINGDOM

## Abstract

The recent blood flow and magnetization transfer (MT) technique termed alternate ascending/descending directional navigation (ALADDIN) achieves the contrast using interslice blood flow and MT effects with no separate preparation RF pulse, thereby potentially overcoming limitations of conventional methods. In this study, we examined the signal characteristics of ALADDIN as a simultaneous blood flow and MT imaging strategy, by comparing it with pseudo-continuous ASL (pCASL) and conventional MT asymmetry (MTA) methods, all of which had the same bSSFP readout. Bloch-equation simulations and experiments showed ALADDIN perfusion signals increased with flip angle, whereas MTA signals peaked at flip angle around 45°−60°. ALADDIN provided signals comparable to those of pCASL and conventional MTA methods emulating the first, second, and third prior slices of ALADDIN under the same scan conditions, suggesting ALADDIN signals to be superposition of signals from multiple labeling planes. The quantitative cerebral blood flow signals from a modified continuous ASL model overestimated the perfusion signals compared to those measured with a pulsed ASL method. Simultaneous mapping of blood flow, MTA, and MT ratio in the whole brain is feasible with ALADDIN within a clinically reasonable time, which can potentially help diagnosis of various diseases.

## Introduction

Radio frequency (RF) pulses used for arterial spin labeling (ASL) cause off-resonance saturation, i.e., magnetization transfer (MT), which confounds the ASL-based blood perfusion measurement. Methods to suppress these MT effects have been proposed for pulsed ASL (PASL) [1–4], continuous ASL (CASL) [5–7], and pseudo-continuous ASL (pCASL) [8, 9]. In nearly all ASL methods, these MT and MT asymmetry (MTA) effects are not considered as meaningful signals.

When MTA is intentionally unsuppressed, its effect can be determined and potentially used for clinical applications [10–12]. The asymmetry of the MT effects arises from the mismatch between centers of bulk water and solid-like macromolecules [6]. Therefore, MTA from macromolecules may provide distinct information under pathologic conditions different from the MT effects [11].

In the absence of a dedicated preparation pulse, blood perfusion and MT effects commonly occur in multiple-slice 2D imaging [13–17]. These 2D inter-slice blood flow and MT effects have been considered confounding factors for a long period of time, however, they may be exploited as methods for imaging perfusion and MT effects. Of late, an imaging technique termed, alternate ascending/descending directional navigation (ALADDIN) was developed for the acquisition of perfusion-weighted (PW) [18] and MTA images [19], based on the inter-slice blood flow and the MT effects, respectively. The inter-slice MT effects can be also used for imaging MT ratio (MTR) in the whole brain within a clinically feasible scan time [20].

Although previous studies have shown feasibility of ALADDIN approaches for PW and MTA/MTR imaging, the signal characteristics of ALADDIN have not been understood well and simultaneous acquisition of ALADDIN PW and MTA/MTR images has not been investigated together. In this study, we theoretically and experimentally investigated the effects of RF power (i.e., flip angle) on ALADDIN PW and MTA signals and their quantitative aspects. We performed Bloch equation simulations to predict labeling efficiency hence to quantitatively map cerebral blood flow in the conventional unit (ml/100g/min) by modifying a continuous ASL model [21], and to simulate MTR asymmetry (MTR_Asym_) signals based on a two-pool MT model [22]. ALADDIN PW and MTA signals were respectively evaluated with pCASL at various post-labeling delay (PLD) times and a conventional MTA method at various off-resonance saturation frequencies, all of which have the same balanced steady-state free precession (bSSFP) readout. Potential advancements and pitfalls of ALADDIN are also discussed.

## Materials and Methods

### Theory

**Fig 1:**
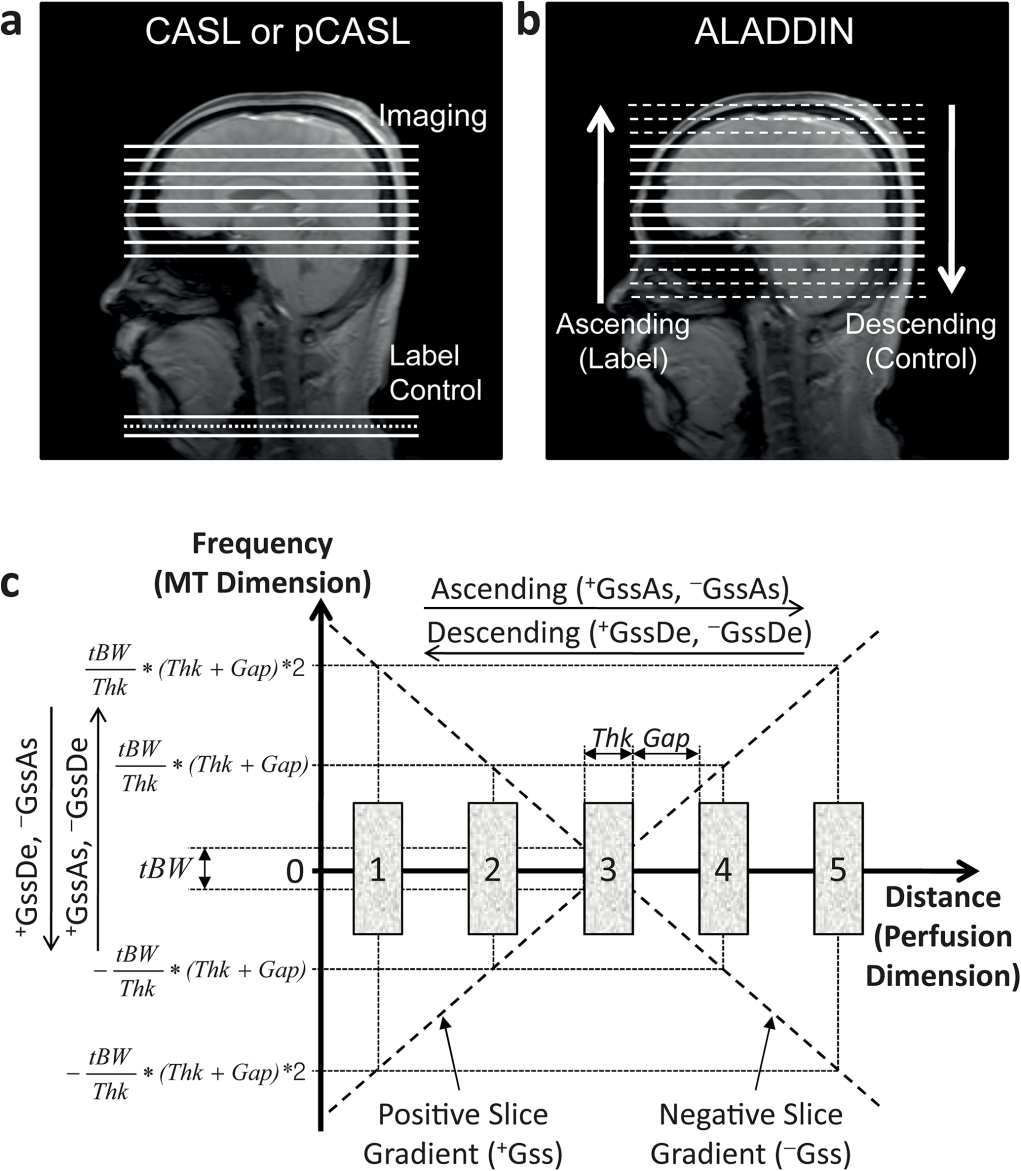
Schematic diagrams for ALADDIN. **a and b**: Diagrams comparing the conventional methods of continuous or pseudo-continuous arterial spin labeling (CASL or pCASL) (**a**) with ALADDIN perfusion-weighted imaging (**b**). **c**: Diagram demonstrating the relationship between the perfusion domain and the MT domain. The horizontal axis represents the physical distance domain, which governs perfusion characteristics (perfusion dimension). The vertical axis represents the offset frequency domain, which governs characteristics of MT asymmetry effects (MT asymmetry dimension). Five slices are considered for ALADDIN signals with the slice of interest to be the third slice. Two diagonal lines represent the slice select gradients with positive and negative polarities, linking the inter-slice perfusion (horizontal) and MT asymmetry (vertical) dimensions. ^*+*^ *GssAs*, ^*+*^ *GssDe*, ^*−*^ *Gss*As, and ^*−*^ *GssDe* represent the imaging with different combinations of positive (^*+*^ *Gss*) / negative (^*−*^ *Gss*) slice select gradients and ascending (*As*) / descending (*De*) slice acquisition orders.

**Fig 2:**
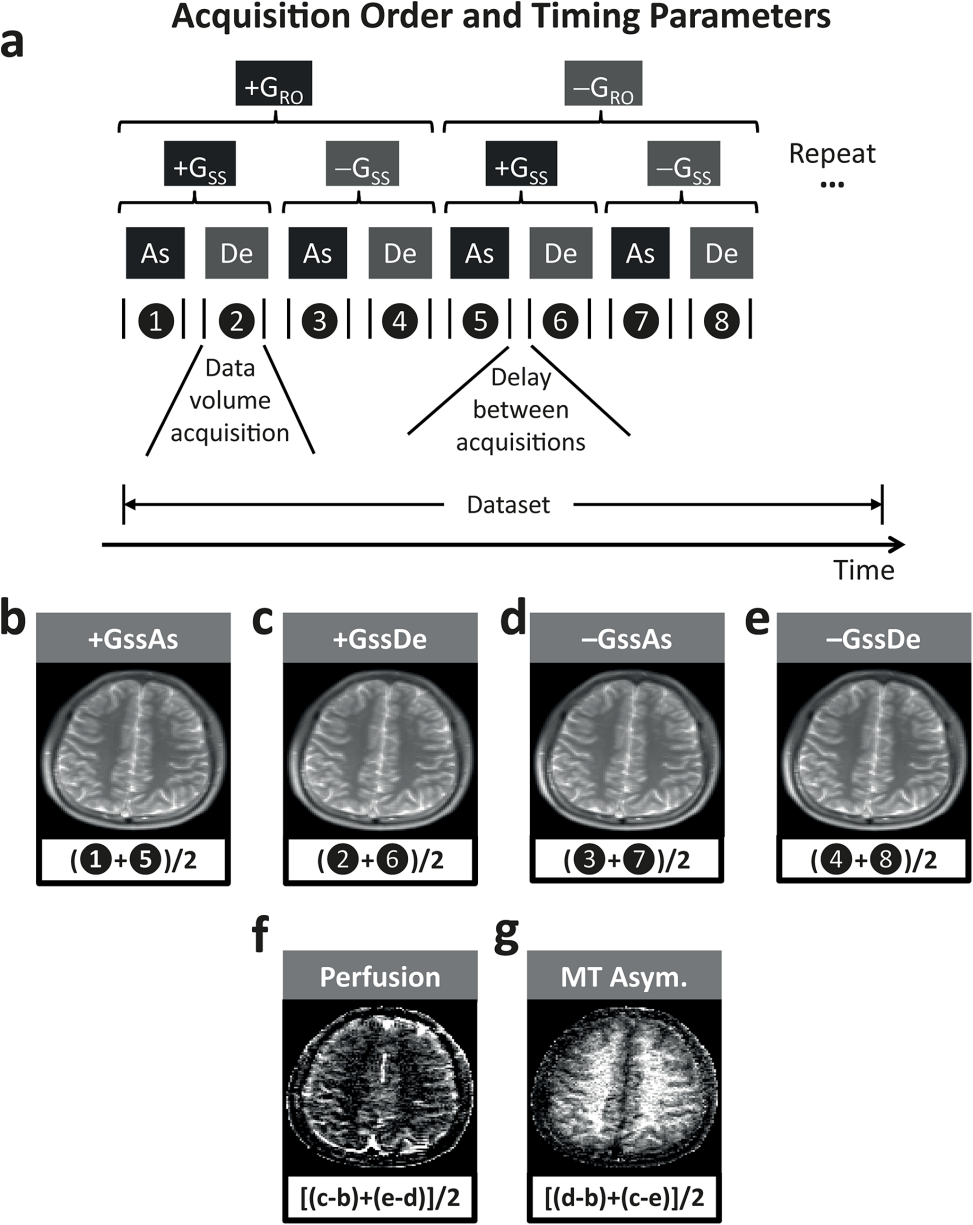
Fundamentals of ALADDIN data acquisition and reconstruction. **a**: ALADDIN acquisition order and timing parameters. The ascending (As) and descending (De) acquisitions were repeated not only with the positive and negative slice-select gradients (^+^G_SS_, ^−^G_SS_) but also with the positive and negative readout gradients (^+^G_RO_, ^−^G_RO_). **b−g**: ALADDIN image manipulation. The images acquired from **b−e** are combined to generate ALADDIN PW (**f**) and MT asymmetry (**g**) images. The terminology “data”, “volume”, “acquisition”, and “delay between acquisitions” are used as explained in this figure throughout the paper.

Perfusion signals in conventional ASL methods are acquired as difference between separate scans with dedicated labeling and control preparations (Fig 1A), whereas those in ALADDIN can be acquired with sequential multiple-slice 2D imaging as difference between separate scans with ascending and descending acquisition orders (with no separate preparation pulse) (Fig 1B). As shown in Fig 1C, the dimension for ALADDIN PW signals (spatial distance dimension along the slice-select gradient, horizontal axis) and that for ALADDIN MTA signals (frequency dimension, vertical axis) are linked with the slice-select gradient. In ALADDIN acquisitions [18, 19], four distinct data acquisition methods (^*+*^ *GssAs*, ^*+*^ *GssDe*, ^*−*^ *Gss*As, ^*−*^ *GssDe*) are identified from the combination of the positive (^*+*^ *G*_*SS*_) or negative (^*−*^ *G*_*SS*_) slice-select gradient, with the ascending (*As*) or descending (*De*) acquisition order (Figs 1 and 2). The readout gradient polarity also needs to be alternated for averaging (Fig 2A), to suppress eddy current contributions [23].

In ALADDIN PW imaging, the acquisition orders corresponding to (ascending) and opposite to (descending) the arterial blood movement work as labeling and control scans, respectively (Fig 1B). Specifically, in order to get percent signal changes (PSC) for the PW signals, datasets of the ascending order (^*+*^ *GssAs* and ^*−*^ *GssAs*) can be subtracted from those of the descending order (^*+*^ *GssDe* and ^*−*^ *GssDe*) as shown in Figs 1C and 2F or as the following Eq 1, where MTA and magnetic field inhomogeneity terms can be suppressed [18].

$$PSC_{PW}=\left\{\left(\;{}^{+}GssDe-\;{}^{+}GssAs\right)+\left(\;{}^{-}GssDe-{}^{-}GssAs\right)\right\}/2/S_{Avg}\times 100\%=\frac{\Delta S_{PW}}{S_{Avg}}\times 100\%$$ (1)

where *∆S*_*PW*_ represents the perfusion difference signal and *S*_*Avg*_ represents the average signal of the four datasets. With approximation of a single long labeling duration and post-labeling delay time (PLD) of 0 for ALADDIN, we can convert the above *PSC*_*PW*_ image into quantitative perfusion map from a continuous ASL model (Eq.17 in [21]) as follows:

$$\begin{array}{l} CBF=\frac{\Delta S_{PW}}{S_{0}}\cdot \frac{\lambda }{2\cdot \alpha \cdot T_{1app}}\cdot \frac{1}{\left(1-\text{exp}(-\tau /T_{1app}\right)}\\ \;=\frac{\Delta S_{PW}}{S_{0}}\cdot \frac{\lambda }{2\cdot \alpha \cdot T_{1app}}\cdot \frac{1}{\left(1-\text{exp}(-\tau_{0}/T_{1app}\right)}\cdot \frac{1}{\sum_{i=1}^{N_{eff}}\text{exp}(-w(i)/T_{1app})} \end{array}$$ (2)

$$\alpha =\frac{1}{2}\cdot \left[\left(M_{Z\;0}^{Blood}(\theta ,TR)-M_{Z\;Label}^{Blood}(\theta ,TR)\right)/M_{Z\;0}^{Blood}(\theta ,TR)\right]$$ (3)

$$\tau_{0}=TR\cdot N_{\;PE}$$ (4)

$$w(i)=\tau_{0}\cdot (i-1)$$ (5)

$$\tau =\tau_{0}\cdot N_{\;eff}$$ (6)

where S_0_ is the MT-free baseline image, *λ* is the brain/blood partition coefficient (90 ml/100g), *T*_*1app*_ is the longitudinal relaxation time of the tissue in the presence of flow and macromolecular saturation, *τ*_0_ is labeling duration from one prior slice (i.e., acquisition time for single slice), *τ* is total effective labeling duration (i.e., total acquisition time for the effective number of prior slices affecting perfusion signals), *α* is the labeling efficiency in one prior slice, *w*(*i*) is the PLD time for blood spins labeled at the *i*-th prior slice, $M_{Z\;0}^{Blood}$ and $M_{Z\;Label}^{Blood}$ are the longitudinal magnetizations of the equilibrium and the labeled blood water protons, respectively, *θ* is the flip angle, *N*_*PE*_ is the total number of phase-encoding (PE) steps per slice, and *N*_*eff*_ represents the effective number of prior slices affecting perfusion signals. As shown in Eq 2, ALADDIN PW signals can be represented either as labeled signals from a single labeling plane with a long labeling duration (*τ*) and PLD time of 0 s, or as sum of labeled signals from multiple prior slices with a constant labeling duration (*τ*_0_) and various PLD times (*w*(*i*) = *τ*_0_ ⋅(*i*−1), 1 ≤ *i* ≤ *N*_*eff*_). The two expressions are mathematically identical, since the geometric series in Eq 2 ($\sum_{i=1}^{N_{eff}}\text{exp}(-w(i)/T_{1app})$) is equal to (1 − exp(−*τ* / *T*_1*app*_))/(1 – exp(−*τ*_0_ / *T*_1*app*_). The labeled blood spins relax back to the original state in both the blood pool (T_1b_) and the tissue pool (T_1app_), but we approximated the effective T_1_ relaxation time unified at T_1app_ for simplicity (the errors introduced from the difference between T_1_ relaxation of blood and tissue are typically small [24]). The transverse magnetization of the labeled blood spins was assumed not to contribute to the perfusion signals in the imaging slice, since the blood T_2_ (< 300 ms) is shorter than the time required for the labeled blood to move into the imaging regions. Furthermore, the above ΔS_PW_ and S_0_ in Eq 2 are assumed to be measured at the beginning of bSSFP acquisition in the slice of interest. However, RF excitations in bSSFP decrease the value of ΔS_PW_/S_0_ up to the k-space center position, which is denoted as “contrast-reduction ratio” throughout the paper. In a previous bSSFP-based perfusion study, it is reported that the contrast-reduction ratio became 0.96 and 0.75 with number of RF excitations of 12 for centric PE order and 128 for linear PE order, respectively, when T_1_ = 1200 ms, T_2_ = 100 ms, and TR = 4.6 ms [25]. In this study, we used centric PE order in most cases to minimize the contrast-reduction ratio, except the flip angle-dependent study for comparison with linear PE order (Table 1). We simulated the contrast-reduction ratio as a function of number of PE steps at various flip angles, which was used to correct the quantitative CBF map as demonstrated later.

**Table 1:** Summary of Experimental Parameters.

	Flip Angle-Dependent Study		Comparison Study		Meningioma Patient	
Readout Seq.	bSSFP		bSSFP		bSSFP	
FA (°)	Variation from 15° to 90° in increments of 15°		60		60	
TR/TE (ms)	4.12/2.05		4.15/2.08		4.15/2.08	
Matrix Size	128 × 128		128 × 128		128 × 128	
FOV	230 × 230 mm^2^		220 × 220 mm^2^		220 × 220 mm^2^	
rBW (Hz/pixel)	673		592		592	
tBW (kHz)	1.07		1.33		1.33	
RF Duration (ms)	1.5		1.2		1.2	
PE Order	Both		Centric		Centric	
No. of Dummy PE Steps	20		30		30	
Number of Subjects (N)	6		6		1	
Additional Settings	Phase OS = 50%		Phase PF = 6/8			
Acq. Method	ALADDIN	MT-FREE	ALADDIN	MT-FREE	ALADDIN	MT-FREE
No. of Slices	15	7	19	13	37 (19+18)	19
Gap (%)	140	N/A	140	N/A	140	20
Inter-Slice Delay (s)	0	8	0	5	0	5
NEX	4	1	8	1	8	1
Acq. Delay (s)	8	N/A	1	N/A	1	N/A
Scan Time per Dataset (min)	2.7	~1	2.9	~1.1	5.7	~1.7
Scan Time per Volume (s)	12.4	~1	10	~1.1	10	~1.7

The parameters rBW represent receiver bandwidth, tBW represent transmit bandwidth, OS represent oversampling, PF represent partial Fourier, and NEX represents number of excitations (averages) for pair-wise subtraction. Image plane = axial, slice thickness = 5 (mm), and dummy PE steps with linearly increasing flip angle = 10 are common imaging parameters for all acquisitions shown in the table.

Similarly, PSC for the MTA signals can be acquired by subtracting datasets of negative inter-slice MT offset frequencies (^*+*^ *GssAs*, ^*−*^ *GssDe*) from those of positive inter-slice MT offset frequencies (^*+*^ *GssDe*, ^*−*^ *Gss*As) as shown in Figs 1C and 2G or as the following Eq 7, where the PW and magnetic field inhomogeneity terms can be suppressed [19].

$$PSC_{MTAsym}=\left\{\left({}^{-}GssAs-{}^{+}GssAs\right)+\left({}^{+}GssDe-{}^{-}GssDe\right)\right\}/2/S_{Avg}\times 100\%=\frac{\Delta S_{MT}}{S_{Avg}}\times 100\%$$ (7)

MTR_Asym_ is typically defined using S_0_ in the denominator and can be converted from the abovementioned PSC_MTAsym_ as follows:

$$MTR_{Asym}(F_{0})=\frac{\Delta S_{MT}(F_{0})}{S_{0}}\times 100\%=PSC_{MTAsym}(F_{0})\cdot \frac{S_{Avg}}{S_{0}}$$ (8)

where *F*_*0*_ represents the inter-slice frequency offset.

The acquisition of S_0_ (i.e., the MT free images) also allows us to get MTR images as follows [20]:

$$MTR=\frac{S_{0}-S_{Avg}}{S_{0}}$$ (9)

The frequency offset of the *i* ^th^ prior slice in the frequency dimension relative to the slice of interest can be expressed as shown in the vertical axis in Fig 1C and also in a generalized form as follows:

$$F_{0}\left(i\right)=\left(tBW/Thk\right)\cdot \left(Thk+Gap\right)\cdot i=tBW\cdot \left(1+Gap/Thk\right)\cdot i$$ (10)

(t*BW*: the transmit bandwidth; *Thk*: the slice thickness, and *Gap*: the inter-slice gap). The above Eq 10 is also valid for negative values of *i*, i.e., future slices for the acquisition. Note that the inter-slice frequency offsets are determined by the ratio of *Gap* to *Thk*, rather than the individual *Gap* and *Thk* values.

### Ethical Considerations

All experiments were approved by the Institutional Review Boards at the University of Pittsburgh and Seoul National University and written informed consent was obtained from all participants.

### Data Acquisition

All experiments were performed on 3T Tim Trio and Verio whole body scanners (Siemens Medical Solutions, Erlangen, Germany) with a body coil for transmission and a 12-element head matrix coil for reception. Total 13 subjects were tested in this study. Various studies were performed at different scan conditions. Scan parameters for the studies including the number of subjects for each study are summarized in Table 1.

#### Flip Angle-Dependent Study

ALADDIN PW and MTA images were acquired as shown in Fig 2A [23]. A long delay time between acquisitions (e.g. 8 s) was used to avoid contributions of any relaxation effects (to make comparisons clear) and also to allow scans of high flip angle (e.g. 90°) considering specific absorption rate (SAR) limitation (note that a long acquisition delay is not necessary for typical ALADDIN acquisitions with flip angles < 90°). We tested these scan conditions with a 4% agarose phantom and confirmed no artifactual signal in both PW and MTA images. As a comparison reference to quantitative ALADDIN PWI, a PASL study [26] was performed using an EPI sequence with TI_1_ = 0.7 s; TI_2_ = 1.8 s; TR = 4 s; matrix size = 64 × 64; field-of-view (FOV) = 230 × 230 mm^2^; number of excitations (NEX) (for pair-wise subtraction) = 30; slice thickness = 5 mm; gap = 7 mm; number of slices = 7; and scan time = 4 min. The labeling slab had 10-cm thickness and 1-cm gap relative to the proximal edge of the imaging slices. For quantitative blood flow mapping with PASL, the effective TI_2_ was adjusted for each slice and the *q* value was assumed to be 1 [26].

#### Comparison Study with Conventional Methods

A separate study was performed to compare sensitivity of ALADDIN PW and MTA imaging to conventional single-slice pCASL [8, 9, 27] and MTA, which were performed using the same bSSFP readout, number of averages, and the same imaging parameters as ALADDIN, with sufficient acquisition delay time (e.g. >5 s) to get rid of any residual signals prior to each measurement. The inter-slice saturation condition of the ALADDIN scan was: pulse width = 1.2 ms; TR = 4.15 ms; and average RF power = 0.94 μT. For pCASL imaging, three images with PLD times of 0, 0.5, and 1.0 s were separately acquired to emulate the perfusion effects respectively equivalent to the first, second, and third prior slices in ALADDIN imaging. The labeling parameters were: RF pulse shape = Hanning window; flip angle = 25°; RF duration = 0.5 ms; inter-pulse delay = 0.92 ms; slice-selective gradient = 6 mT/m, total tagging duration = 1500 ms; distance between labeling and imaging plane = 8 cm; and balanced tagging scheme. For conventional MTA imaging, a pulse train of 75 Gaussian pulses were used for the MT saturation, with pulse width = 20 ms, inter-pulse delay = 20 ms, flip angle = 578.4°, total saturation duration = 3 s, and average RF power = 0.94 μT corresponding to the average RF power of ALADDIN. The pre-saturation pulse was applied with six different off-resonance irradiation frequencies of ±3200 Hz, ±6400 Hz, and ±9600 Hz, to generate three different MTA images equivalent to the MT effects from the first (±3200 Hz), second (±6400 Hz), and third (±9600 Hz) prior slices in ALADDIN imaging. Contributions of the three prior slices were dominant in ALADDIN MTA, as shown in a previous study [19] and later in this study with simulations and experiments.

#### Application to a Meningioma Patient

ALADDIN was performed on a meningioma patient. Two datasets with number of slices = 18 and 19 were separately acquired and then interleaved to cover the whole brain regions in the conventional gap value (20% of the slice thickness in this study), yielding a scan time of 5.7 min. MT free images (*S*_*0*_) were additionally acquired with: 5 s inter-slice delay time; the positive slice-select gradient; ascending order; and scan time = ~1.7 min. For comparison, fluid attenuated inversion recovery (FLAIR) imaging was performed with scan parameters of: TR = 5000 ms; TE = 93 ms; echo train length = 16; FOV = 220 × 178 mm^2^; matrix size = 256 × 208; slice thickness = 5 mm; number of slices = 25; flip angle = 130°; NEX = 1; and scan time = ~2.4 min.

### Data Processing

ALADDIN PW and MTA images were reconstructed based on the above Eqs 1 and 7, as described previously (Fig 2F and 2G) [18, 19, 23]. Motion correction [28] was applied to each dataset with different slice-select gradient polarity. PW and MTA images were calculated from four datasets that were corrected for motion and averaged.

Whole gray matter (GM) and white matter (WM) regions and a region with no brain signal were manually segmented from the center slice of the baseline images. Quantitative cerebral blood flow (CBF), MTA, and PSC values were calculated within the regions of interest. Signal-to-noise ratios (SNRs) of PW and MTA images were calculated from GM and WM, respectively, in their subtraction images, as mean of the signals divided by the background noise level (i.e., standard deviation of the signals in the region with no brain signal). This spatial SNR was not corrected for averaging.

To further evaluate the differences in different perfusion imaging methods (i.e., ALADDIN and pCASL), a standard bootstrapping procedure [29] was adopted to simulate calculation of temporal SNR. Specifically, 1000 bootstrap samples of tag and control images were sampled using non-parametric re-sampling with replacement from each subject data (N = 6) to generate artificial time-series of perfusion signal. The mean and standard deviation of the resulting perfusion subtraction signal were then used to calculate the temporal SNR on a voxel-wise basis.

### Computer Simulations

Computer simulations were performed using Bloch equations to investigate ALADDIN PW and MTA signals as a function of flip angle. Magnetizations of labeled blood spins were simulated under an assumption that they experience RF excitations in a prior slice, move through the following slices with a constant blood velocity, and then perfuse at the slice of interest. The range of blood velocities investigated for the simulation was from 2 cm/s to 30 cm/s with 1 cm/s steps. Since the propagation speed of ALADDIN acquisitions was ~1.5 cm/s under our experimental condition (i.e., thickness = 5 mm, gap = 7 mm, and time to get one slice = ~0.8 s) which is much slower than the typical arterial blood speed used for the simulation, blood spins were assumed not to experience RF excitations from more than one prior slices (note that blood spins experience RF excitations from more than one prior slices only when blood velocity is similar to the slice propagation speed of ALADDIN). The phase evolution angle ranged from −180° to 180° with 12° step and RF phase-cycling angle was set to 180°. To minimize variations due to oscillatory magnetizations [18], the longitudinal magnetizations of labeled blood spins ($M_{Z\;Label}^{Blood}(\theta ,TR)$) were determined as average of two cases of even and odd number of excitations within one prior slice for each blood velocity. Blood labeling efficiency (*α*) was calculated following Eq 3 as a function of blood velocity, phase evolution angle, flip angle, and TR values. For the quantification of CBF maps, blood labeling efficiency was eventually determined by averaging magnetizations of labeled blood spins from all the blood velocities and the phase evolution angles. The longitudinal magnetization of unlabeled blood spins was assumed to be 1 ($M_{Z\;0}^{Blood}\;(\theta ,TR)=1$) regardless of off-resonance frequencies, and thus the labeling efficiency was not artificially enhanced by lower baseline bSSFP signal intensities at off-resonance frequencies. The contrast-reduction ratio was simulated with Bloch equations [30] with GM *T*_1_ and *T*_2_ of 1830 ms and 99 ms [31]. Cerebral blood flow values were calculated following Eq 2 with *T*_1app_ = 1.5 s.

The amount of inter-slice MT effects at both positive and negative frequency offsets were separately simulated for WM using a two-pool MT model (only WM was simulated for simplicity) [22]. A Runge-Kutta method was used to solve the differential equations of Eq 9−12 in the reference [22]. The cumulative MT effects in edge slices were simulated as a function of number of prior slices and found to reach a steady-state condition at a certain number of slices, as demonstrated later. By setting this steady-state MT as an initial longitudinal magnetization, magnetization changes at a slice of interest were simulated as a function of PE step with WM *T*_1_ and *T*_2_ of 1084 ms and 69 ms [31]. Finally, *PSC*_MTAsym_ and *MTR*_Asym_ values for the simulation were calculated following Eqs 7 and 8.

## Results

**Fig 3:**
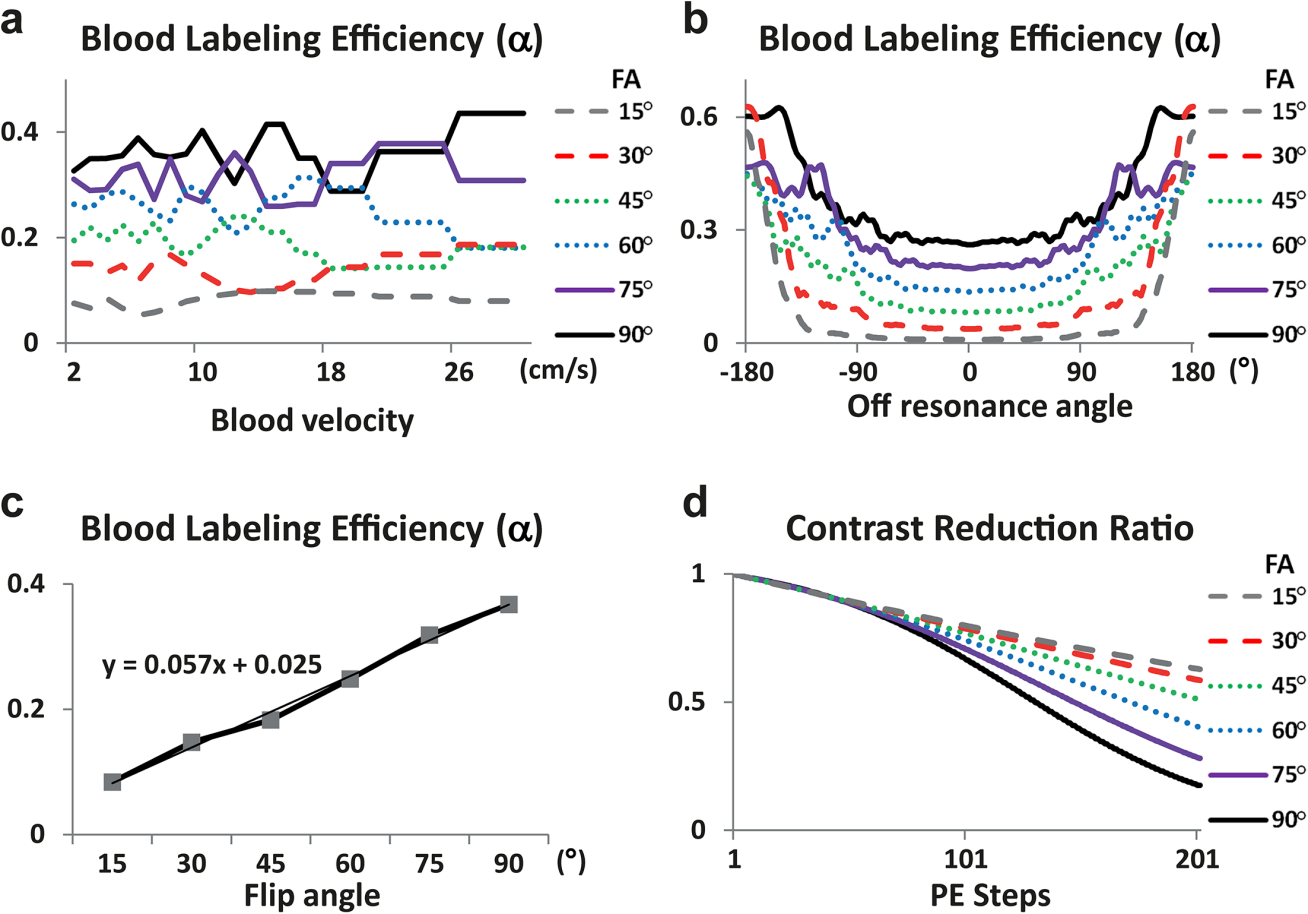
Simulation results for blood labeling efficiency (α) and contrast-reduction factor in ALADDIN PW imaging. **a-c**: Simulation results of blood labeling efficiency at various blood velocities (**a**), off-resonance phase angles (**b**), and flip angles after averaging over blood velocities and off-resonance phase angles (**c**). **d**: Simulation of ratio of reduction in contrast as a function of PE steps. The simulations were performed by numerically solving Bloch equations for bSSFP sequence.

Fig 3 shows the blood labeling efficiency predicted using computer simulations at various scan conditions. The blood labeling efficiency varied with flow velocity (Fig 3A): the standard deviation of the labeling efficiency normalized with its mean (across flow velocities) was 14%, 20%, 17%, 17%, 12%, and 12%, when flip angle was 15°, 30°, 45°, 60°, 75°, and 90°, respectively. The labeling efficiency was higher for off-resonance flow spins than on-resonance flow spins (Fig 3B). The enhancement of blood labeling efficiency for off-resonance spins was relatively higher at lower flip angles (Fig 3B). Overall blood labeling efficiency increased with flip angle (Fig 3C). The perfusion contrast decreased with RF excitations faster at higher flip angles, which was not noticeable until the number of PE steps reached ~100 (Fig 3D). This simulated contrast reduction ratio was used for the correction of quantitative blood flow values.

**Fig 4:**
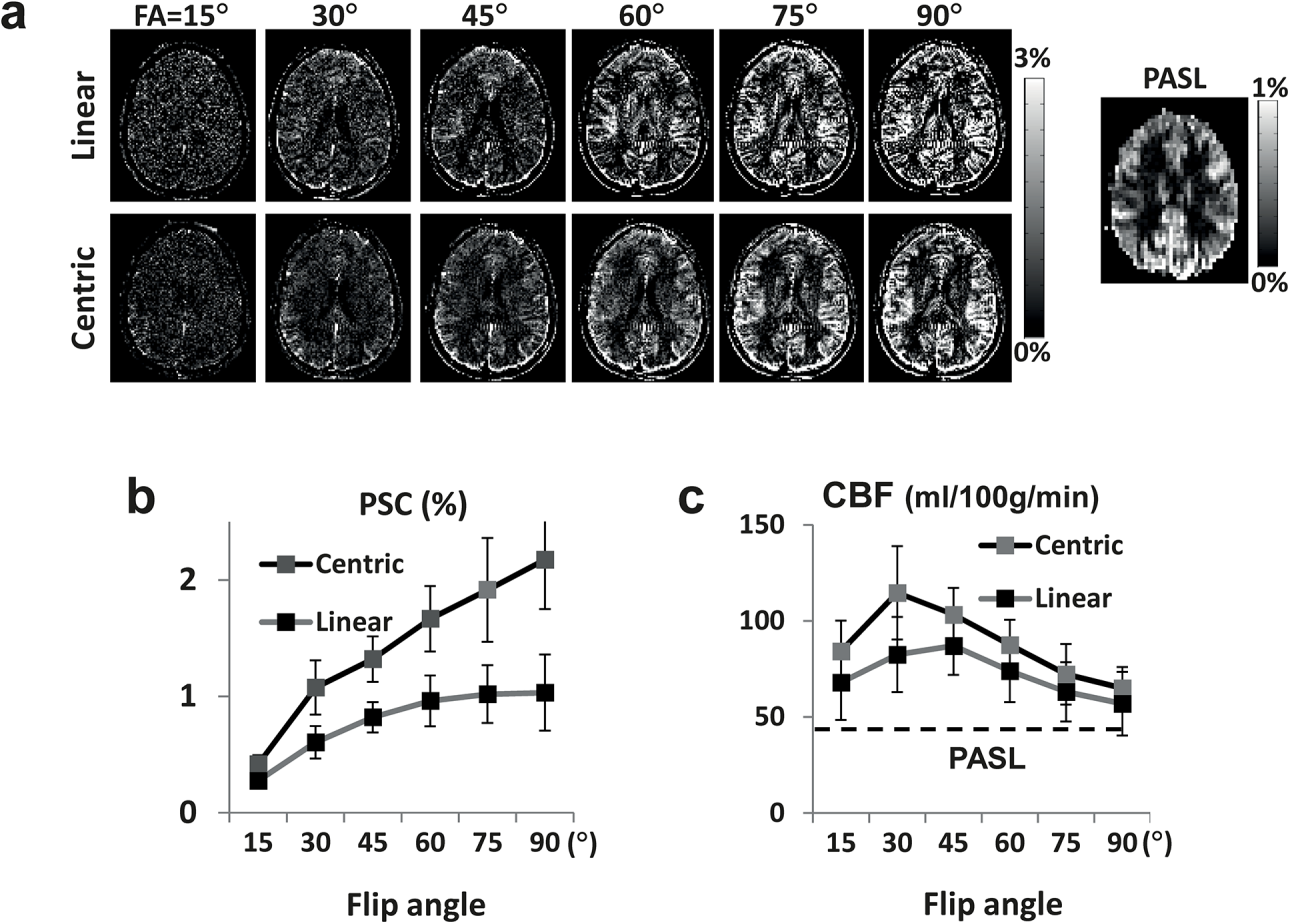
ALADDIN PW signals as a function of flip angle. **a**: PW images displayed as PSC maps at various flip angles for linear and centric PE orders of ALADDIN and for PASL. **b**: PSC of ALADDIN PW signals measured in GM as a function of flip angle. **c**: Quantitative perfusion signals measured with ALADDIN as a function of flip angle. The broken lines represent those from PASL. Scan time for ALADDIN and PASL was 2.7 min and 4 min, respectively.

Experimental results for ALADDIN PW imaging are shown for one representative subject (Fig 4A) and averaged signals over all the subjects tested (Fig 4B and 4C). The PW signals continued increasing with flip angle up to 90°, in agreement with the simulation results (Fig 3C). The centric PE order showed overall higher PW signals than linear PE order (Fig 4B). The mean CBF values from ALADDIN were higher than those from PASL (40±11 ml/100g/min) (Fig 4C) at all flip angles, with higher CBF values at centric PE order than linear PE order. Standard deviation of CBF values was higher at lower flip angles presumably due to low SNR.

**Fig 5:**
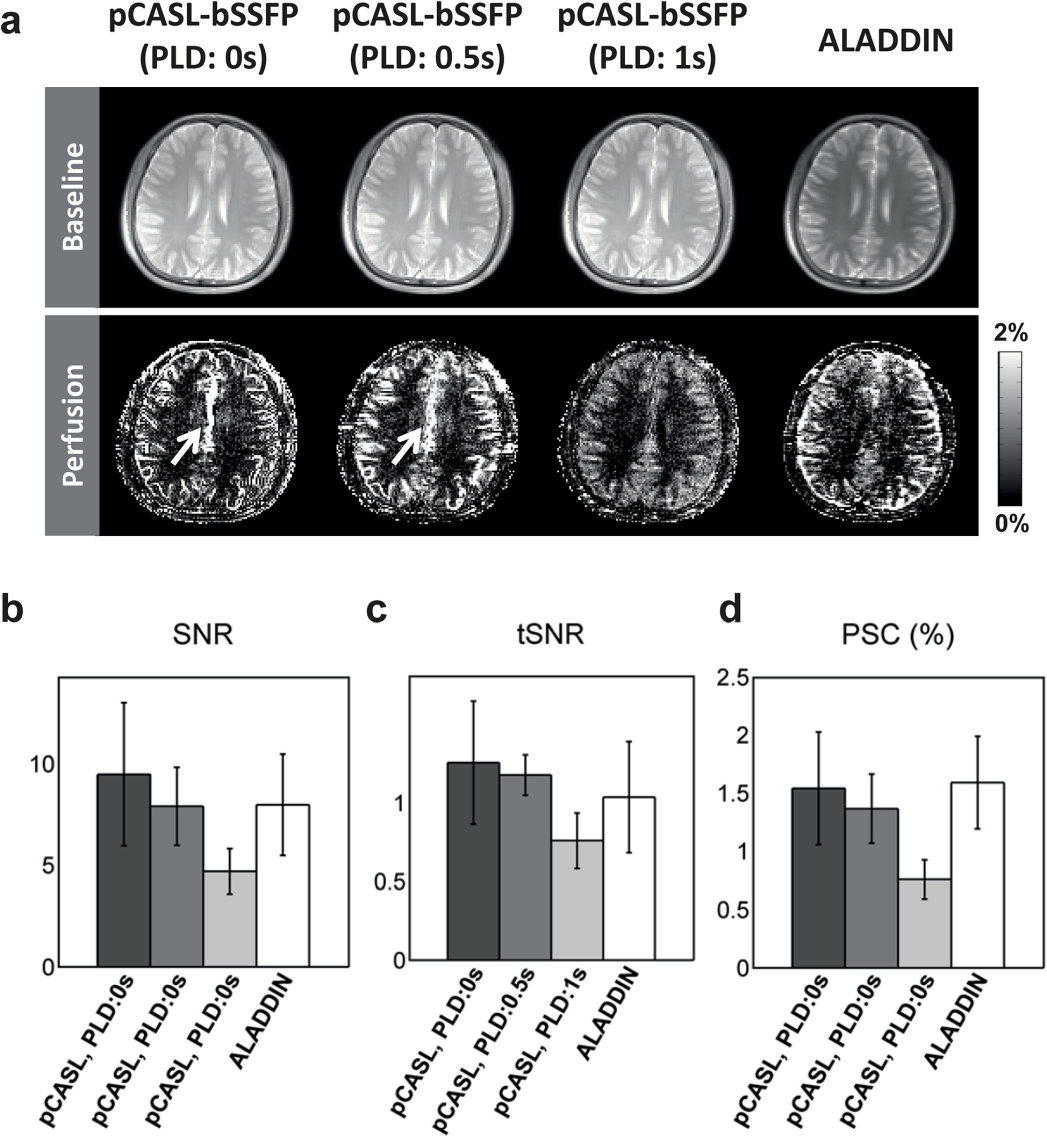
Comparison of ALADDIN and pCASL perfusion imaging. **a**: Baseline and PW images of ALADDIN and pCASL images with PLD times of 0 s, 0.5 s, and 1 s from a representative subject. The PW images are displayed in PSC. The arrows represent strong blood signals in pCASL with PLD of 0 s and 0.5 s, which were not observed in ALADDIN. **b and c**: Spatial SNR (**b**) and temporal SNR (**c**) of ALADDIN and pCASL PW signals measured in GM (N = 6). The temporal SNR was simulated for ALADDIN and pCASL PW signals from 1000 bootstrap samples (N = 6). Spatial SNR was not corrected for averaging (number of average = 8). **d**: Percent signal changes (PSC) of ALADDIN and pCASL PW signals measured in GM (N = 6). bSSFP readout with flip angle of 60° was used for imaging in all methods.

Fig 5 shows the experimental results for ALADDIN and pCASL-bSSFP with different PLDs for one representative subject (Fig 5A) and averaged signals over all the subjects tested (Fig 5B–5D). As PLD increased, the pCASL perfusion signals decreased but were more localized at tissue regions rather than blood vascular regions (Fig 5A). Intensities and spatial patterns of ALADDIN PW signals were comparable to those of sum of the three perfusion images acquired at different PLD times (Fig 5). The measured SNR and PSC of ALADDIN were comparable to those of pCASL-bSSFP. The strong blood signals in the middle of the brain (between hemispheres) observed in pCASL with PLD 0 s and 0.5 s (arrows in Fig 5A) were not observed in ALADDIN, which was consistent for all the subjects tested. Source of this observation is not clear, and we attribute this to vascular anatomy.

**Fig 6:**
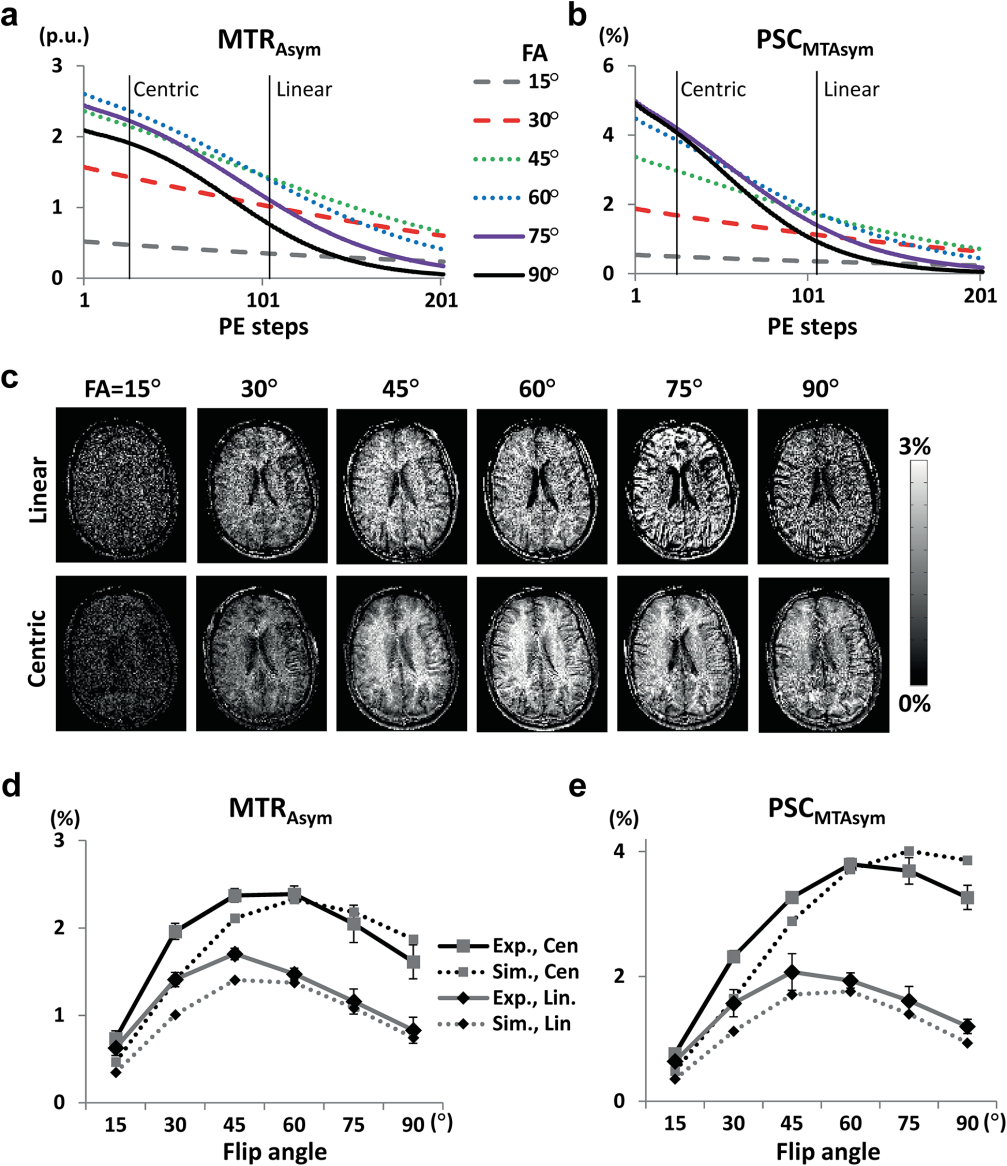
Simulation and experimental results for MTA signals. **a and b**: MT signals were simulated as both MTR_Asym_ and PSC_MTAsym_. MTA signals are calculated as the signal difference between positive and negative MT frequencies divided by MT-free images (i.e. MTR_Asym_) (**a**) and MT-weighted images (i.e. PSC_MTAsym_) (**b**). The vertical lines in the plot indicate the number of phase-encoding steps required to reach the k-space center including the dummy PE steps. **c:** Experimental results for MTA acquired with centric and linear phase-encode orders at various flip angles from a representative subject. Images are displayed as MTR_Asym_. **d and e**: MTR_Asym_ and PSC_MTAsym_ for the experimental results from all the subjects tested (N = 6) (solid lines) in comparison with the simulation results (dotted lines). The black and gray lines indicate centric and linear phase-encode orders, respectively, as indicated.

According to the MT simulation, initial differences in longitudinal magnetizations between positive and negative MT frequencies was the highest at flip angle 60° and decayed with RF excitation faster at higher flip angles (Fig 6A and 6B). In contrast to PW signals, MTR_Asym_ signals peaked at flip angle around 45°−60° (Fig 6C−6E), with similar trends between the simulation and experimental results. The subtle differences between simulation and experiment are presumably due to inaccurate assumption of WM properties (e.g. T_1_, T_2_), magnetic field inhomogeneity, and partial volume effects. PSC peaked at slightly higher flip angle (60°) than MTR_Asym_ (45°) for centric PE order (Fig 6D and 6E), due to higher MT effects at higher flip angles (more signal reduction in the baseline images). Linear PE order peaked at lower flip angle than the centric PE order for PSC (Fig 6E), due to the faster signal decay with PE steps at higher flip angle (Fig 6B). Overall centric PE order provided higher MTA signals than linear PE order.

**Fig 7:**
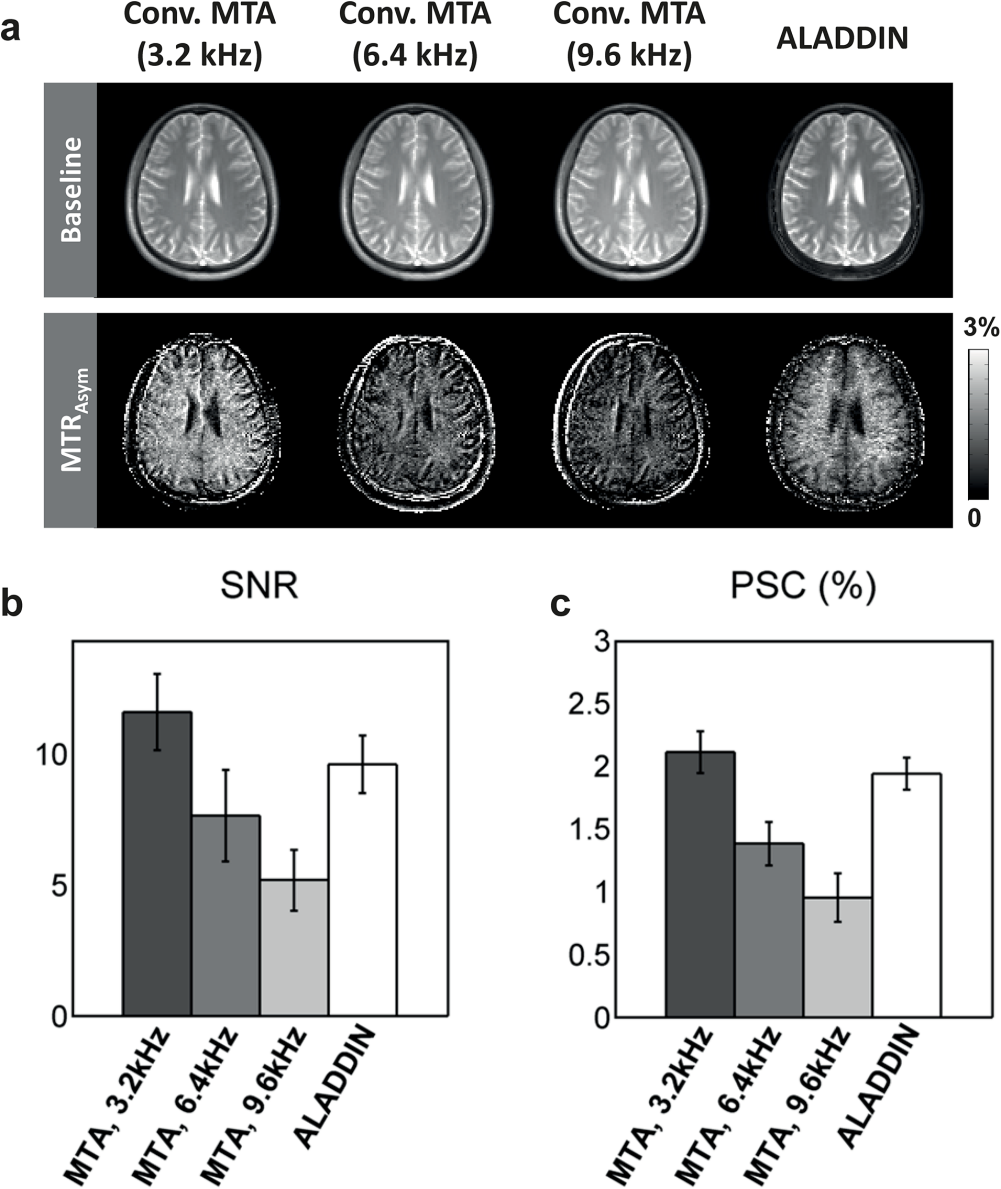
Comparison of ALADDIN MTA to conventional MTA method. **a**: Baseline and MTA images acquired using conventional MTA method with MT effects equivalent to ALADDIN first prior slice (± 3200 Hz), second prior slice (± 6400 Hz), and third prior slice (± 9600 Hz) and those acquired using ALADDIN from a representative subject. The MTA images are displayed in percent signal change (PSC). **b and c**: SNR (**b**) and PSC (**c**) of ALADDIN and conventional MTA images measured in WM (N = 6). bSSFP readout with flip angle of 60° was used for imaging in all methods.

Fig 7 shows the experimental results for ALADDIN and conventional MTA images with different offset irradiation frequencies for one representative subject (Fig 7A) and averaged signals over all the subjects tested (Fig 7B and 7C). Visually, MTA signals were stronger in WM for all images (Fig 7A). MTA from the conventional method showed decreasing signal intensity for increasing offset irradiation frequencies. The SNR and PSC values of the conventional MTA method with MT effects equivalent to ALADDIN first (±3200 Hz), second (±6400 Hz), third (±9600 Hz) prior slices, and those for ALADDIN are shown in Fig 7B and 7C. Overall, intensities and spatial patterns of ALADDIN MTA signals were similar to those of the conventional MTA image acquired with offset frequency corresponding to the ALADDIN first prior slice (i.e., ±3200 Hz).

**Fig 8:**
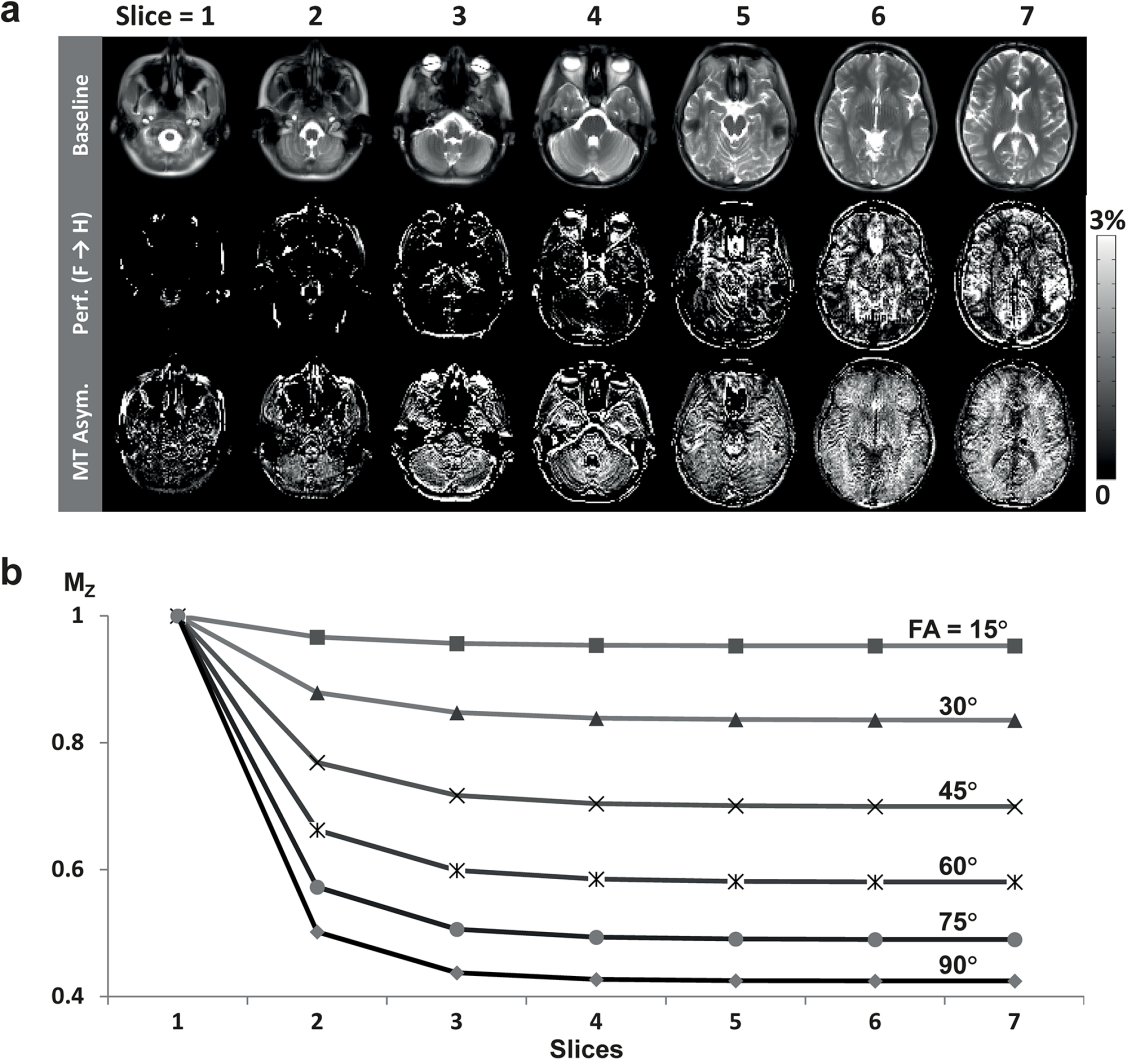
Transient (cumulative) MT effects in edge slices. **a**: Edge slices for baseline, PW, and MTA signals. The PW and MTA signals are displayed in PSC. The data were acquired with centric PE order and flip angle of 90°. **b**: Transient (cumulative) MT effects in edge slices simulated at various flip angles. The transient MT effects are in steady-state from the 5^th^ or 6^th^ slices, i.e., number of dummy slices for PW imaging is 4−5. See text for details.

Effects of transient (cumulative) MT effects in edge slices are shown for a representative subject (Fig 8A) and for the simulation (Fig 8B). PW signals were not visually observable until around the 5^th^−6^th^ slice in the axial ALADDIN PW scan (middle row in Fig 8A). According to the MT-related simulations, the differences between the longitudinal magnetization of the 2^nd^, 3^rd^, 4^th^, 5^th^, and 6^th^ edge slices and the steady-state longitudinal magnetization (e.g. 9^th^ edge slice) became less than 8.3%, 1.9%, 0.5%, 0.1%, and 0.0%, respectively, of the initial magnetization (Fig 8B). Since ALADDIN PW signals are ~1% of baseline intensity, the results indicate that the ALADDIN PW signal would be out of transient cumulative MT effects from the 5^th^−6^th^ slice, which agreed well with the experimental results (middle row in Fig 8A). In contrast to the PW signals, strong positive MTA signals were observable from the 2^nd^ edge slice (bottom row in Fig 8A), due to the inclusion of both ascending and descending acquisitions in each subtraction term for MTA (Eq 7).

**Fig 9:**
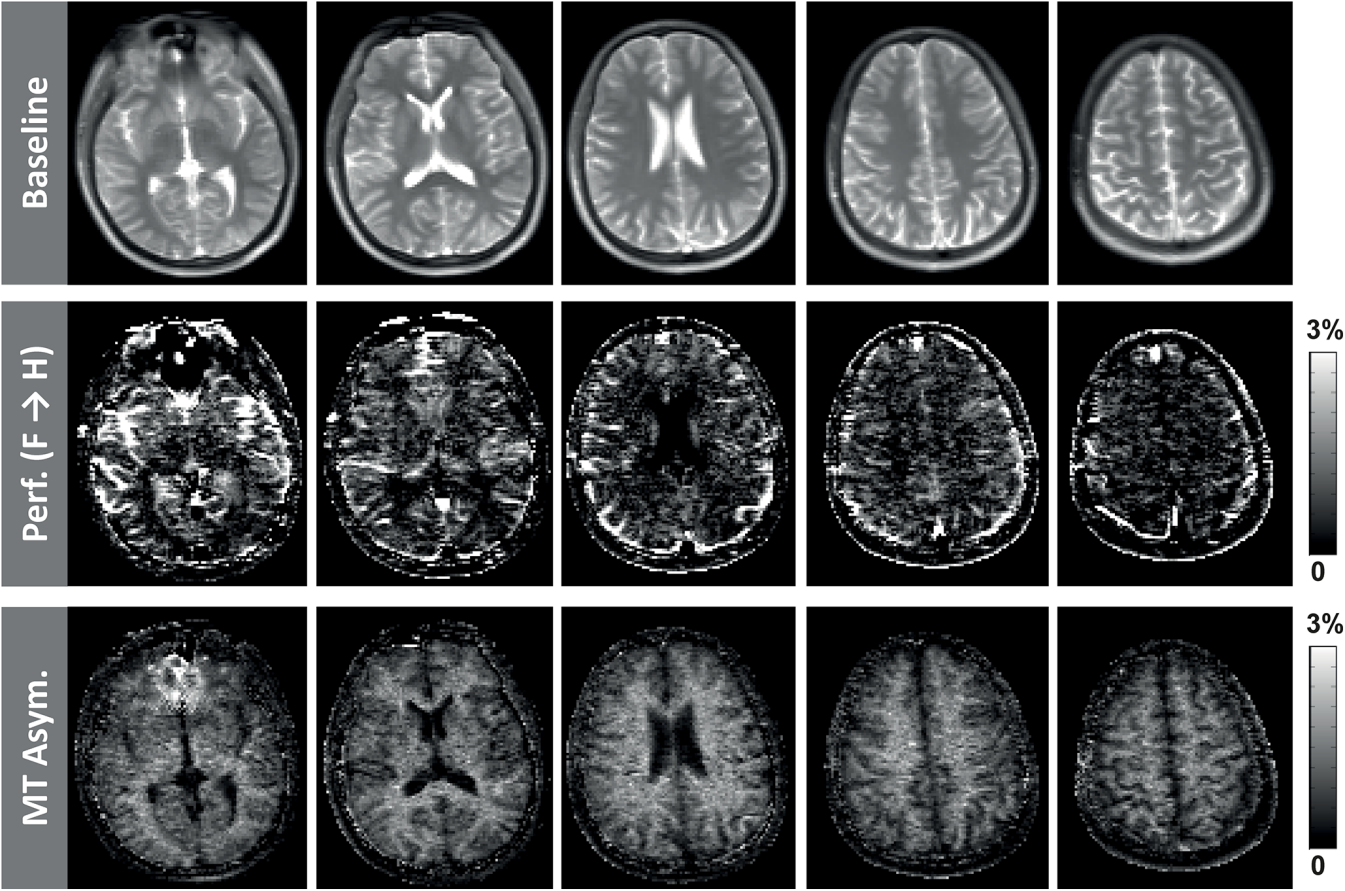
Representative ALADDIN baseline, perfusion, and MT asymmetry acquired simultaneously in a normal volunteer. phase-encoding order = centric, flip angle = 60°, and scan time = 2.9 min.

**Fig 10:**
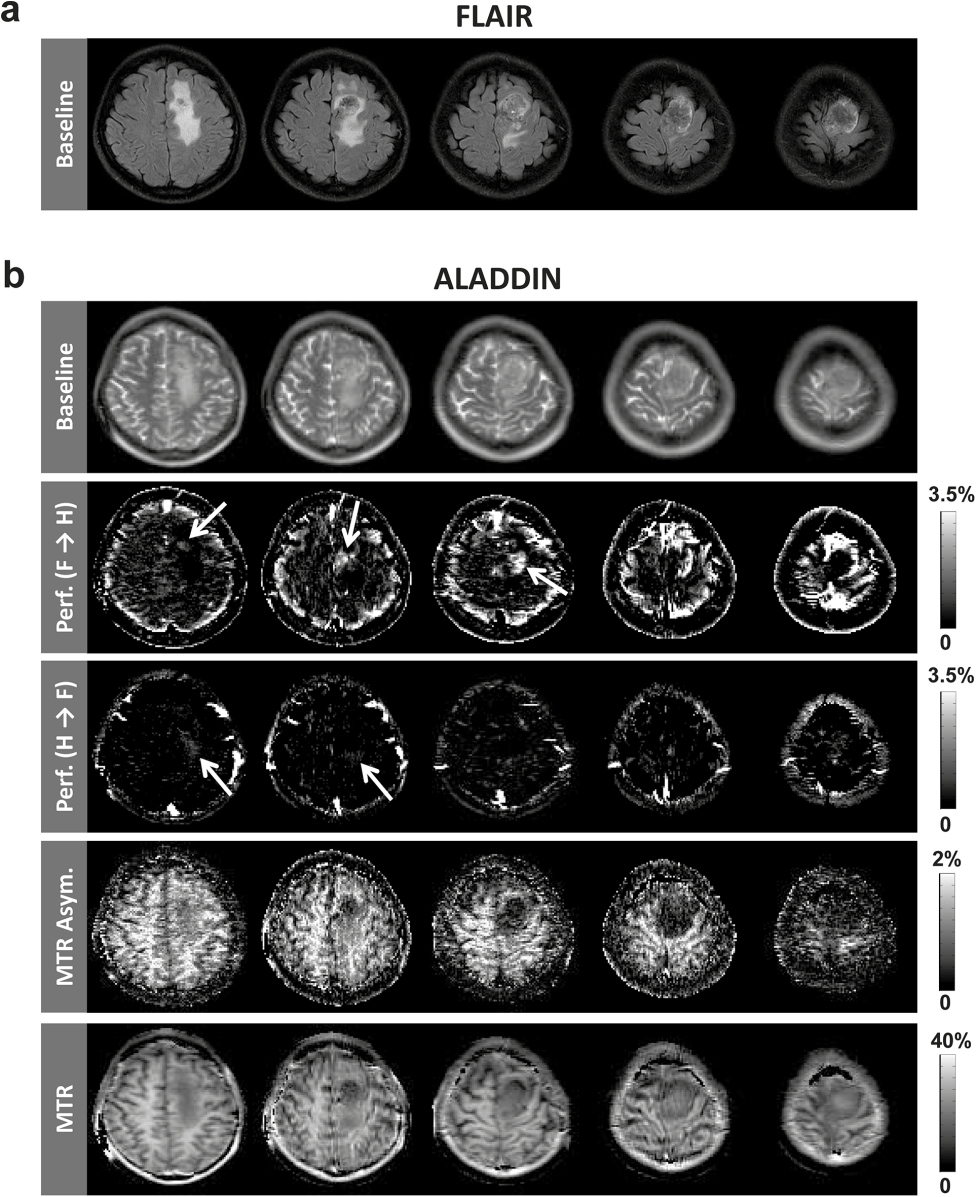
Clinical demonstration of simultaneous mapping of cerebral blood flow, MTR_Asym_, and MTR with ALADDIN. **a:** FLAIR images. The total scan time was ~2.4 min. **b:** ALADDIN images acquired from a brain tumor patient. The arrows indicate heterogeneous blood flow directionality in the tumor region. The data were acquired with centric PE order and scan time was ~5.7 min for ALADDIN acquisition and ~1.7 min for acquisition of MT-free images (total ~7.4 min).

Fig 9 shows representative ALADDIN baseline, PW, and MTA images simultaneously acquired with flip angle 60°, centric PE order, and scan time of 2.9 min. The blood flow signals (middle row in Fig 9) were mostly observed in GM regions and also in some WM regions, and the hyper-intense regions were presumed to be blood vessels. MT asymmetry showed stronger signals in WM than in GM, and also showed heterogeneous signals within the WM regions (e.g. hyper-intense and hypo-intense spots) throughout the brain regions (bottom row in Fig 9), which could not be easily assessed in the corresponding baseline images (top row in Fig 9). The experimental results of ALADDIN for the brain tumor patient are shown in Fig 10. ALADDIN perfusion images showed heterogeneous blood flow directions in the tumor region, as indicated by arrows in Fig 10B (e.g. some portions flowed from feet-to-head (“F→H”) and other regions from head-to-feet (“H→F”)). Overall the tumor region showed high vascularity in ALADDIN perfusion maps. ALADDIN MTA and MTR images showed distinct signal characteristics in the brain tumor regions, different from the FLAIR images and also different from each other.

## Discussion

Multi-sectional PW and MTA images in the whole brain could be simultaneously acquired by the ALADDIN technique with a relatively short scan time (~5.7 min) and were quantitatively mapped or modelled by additionally acquiring MT-free images (~1.7 min). Note that number of acquisitions need not be multiple of 8, as long as it is ≥ 8. PW signals tended to increase with flip angle, while MTA signals peaked at around a flip angle of 45−60°. These trends for PW and MTA signals were in agreement with our simulation results. Nevertheless, the advantage of using high flip angles should be carefully balanced by potential increases in SAR levels.

The ALADDIN SNR and PSC were comparable to those of conventional techniques under the same experiment condition (Figs 5B–5D and 7B and 7C). SNR of ASL is affected not only by number of averages but also by SNR of the baseline images, therefore, the SNR per unit time should be considered by taking into account both of these factors. The high sensitivity of ALADDIN signal may be partly accounted by the usage of bSSFP readout providing the highest SNR per unit time [32, 33] and by the employment of phase oversampling which increases the labeling duration and eventually the SNR of the baseline images. Although a single-shot EPI with high number of averages was frequently used for ASL in the past, the 3D segmented approaches such as 3D GRASE or 3D multiecho (RARE) stack of spiral are recommended as the readout sequence by the ASL community recently, because of high SNR and spatially uniform post labeling delay times [24]. These 3D segmented scans allow smaller number of averages while improving SNR of the baseline images through increased 3D volume, i.e., volume averaging effects. In terms of the number of averages, ALADDIN with 2D bSSFP is intermediate between 2D EPI and 3D sequences used for conventional ASL. The improved SNR of the baseline images through volume averaging effects counteracts the smaller number of averages in these 3D scans. The increased SNR of the baseline images in ALADDIN also counteracts the smaller number of averages, similar to the 3D segmented scans.

Because of phase-alternating nature of bSSFP, it is anticipated that the labeling efficiency of ALADDIN PW imaging might be low. However, we have observed relatively high PSC and SNR in ALADDIN PW images, comparable to those of conventional technique (Fig 5B–5D). Regarding the labeling part, four factors may account for this. One is existence of multiple labeling planes close to the imaging plane (e.g. labeling plane offset of 12, 24, 36 mm for first, second, third prior slices in ALADDIN compared to ~80mm used in pCASL). Another is the long labeling duration (e.g.*τ* ≥ 4 s used in ALADDIN compared to ~1.5 s used in pCASL), as shown in Eq 2. The third is the high signal enhancement of off-resonance flow spins in bSSFP (Fig 3B), due to heterogeneity of signals in off-resonance regions [30, 34]. Flow spins can deviate from on resonance due to variations in resonance frequencies across slices hence feeding arteries, gradient imperfections, etc. A lot of gradients from the incidence of labeling of a flow spin to the time point of imaging may accelerate randomization of its phase in ALADDIN. Lastly, short PLD time due to short traveling distance results in relatively low signal loss (e.g. inter-slice delay time of 0 s used for ALADDIN in this study compared to ~1.5 s used in pCASL), which can account for the relatively high PSC and SNR but also high intravascular arterial signals in ALADDIN perfusion images (Fig 5). ALADDIN showed overall labeling effects comparable to that of pCASL (Fig 5), supporting these notions. Nonetheless, further studies are necessary for more complete understanding of ALADDIN ASL signals.

Implementing interslice delay may suppress the intravascular signals for ALADDIN. In case of the pCASL sequence, PLD > 1 s is necessary for the distance between the imaging and the labeling planes of ~8 cm. ALADDIN has the distance between the labeling and imaging planes of 0.7 cm for the first prior slice, indicating that the arterial transit time is almost negligible. Since it takes ~0.5 s for the labeled arterial blood to exchange with interstitial water in the tissue regions [35], ALADDIN with interslice delay of 0.5 s may suppress intravascular signals at the cost of increase in scan time by roughly 1−2 min under our experimental conditions. Thus, a new strategy for ALADDIN may be necessary to increase interslice delay while maintaining the total scan time.

ALADDIN is different from conventional perfusion or MTA imaging techniques in that it replaces the long preparation pulses required for labeling/MT saturation with a sequential imaging of multiple slices, allowing for both labeling/MT saturation and data acquisition to occur at the same time which eventually decreases total scan time. However, currently ALADDIN requires dummy slices at both ends of the imaging slices to allow for steady-state of the inter-slice MT effects, which reduces its efficiency to {(# of imaging slices) − (# of dummy slices)} / (# of imaging slices). Additionally, if two ALADDIN scans with odd and even numbers of slices are separately acquired and then interleaved to cover whole brain as in the case of this study, efficiency is further reduced. One potential method to decrease the number of dummy slices is employing techniques such as variable flip angle(s) for edge slices to achieve steady-state quicker. Another simple solution to compensate for the loss of efficiency of the proposed technique is to increase the number of imaging slices, since the number of dummy slices is usually fixed to a few slices. The efficiency of the technique can be further improved by improving the RF profile to image whole brain region in one scan. This approach, however, may increase the sensitivity of the technique to undesired cerebrospinal fluid signals and thus should be used with caution.

The results from the meningioma patient imply potential clinical usefulness of ALADDIN. Assessment of physiological and metabolic information in tumor regions is often challenging, possibly due to the uniqueness of the pathological condition along with the limitations in the amount of time to perform multiple scans. ALADDIN has potential for overcoming these limitations, and even may become advantageous over conventional imaging techniques in certain clinical situations. For example, the patient study showed tumor located at the most superior brain region, which may become problematic for conventional ASL techniques due to the longer traveling time for the labeled blood spins to move into the region of interest. ALADDIN perfusion imaging is not affected by tumor locations in the brain (Fig 10B), since labeling is performed in contiguous slices from the imaging plane (Fig 1B). Also, ALADDIN PW images show heterogeneous blood flow directions in the tumor region (exemplified with arrows in Fig 10B), a feature not easily assessed with conventional perfusion imaging methods. Different heterogeneous blood flow information can be also obtained by simply changing the scan direction for ALADDIN imaging [18]. Additionally, MTA and MTR images show similar, but distinct information in the tumor region (Fig 10B). Both MTA and MTR images may reflect different unique metabolic information under pathological condition, since the former represents the offset of center frequencies between the free water and bound pools and the latter is about the strength of the MT effects overall (i.e. the degree of MT saturation). As shown in the previous study, ALADDIN MTR in WM was ~30%, a value similar to that of the conventional method, and the results were supported well by simulation based on two-pool MT model [20]. Altogether, ALADDIN imaging has the potential to provide unique clinical information in a single simultaneous acquisition.

ALADDIN PW signals corresponded to a mixture of perfusion signals from multiple prior slices (i.e., multiple PLD times) (Fig 5), whereas ALADDIN MTA signals were mostly from the first prior slice (Fig 7). This discrepancy between ALADDIN PW and MTA signals regarding contributions of prior slices is ascribed to the facts that delay times are needed for the labeled blood spins to move into tissue regions for the PW imaging and that MT effects directly occur in the tissue regions and start to decay following the delay times for MTA imaging.

The increased delay between acquisitions (8−10 s) in this study (which was ~2 s in the previous studies [18, 19]) did not affect the number of edge slices for ALADDIN PW and MTA imaging. The results suggest that the cumulative MT effects in the edge slices are the dominant factor determining number of edge slices rather than incomplete *T*_1_ recovery between successive acquisitions (Fig 8). As long as the scan protocol is within the SAR limitation, therefore, the delay between acquisitions can be reduced to reduce total scan time.

The bSSFP readout has been demonstrated to be an effective labeling scheme for PASL and pCASL [25, 27]. Centric PE order provided higher signals than linear PE order for both perfusion and MTA (Figs 4 and 6), because property of the k-space center portion determines the image contrast which decays with the RF excitations (Fig 3D). Centric PE order, however, is potentially sensitive to transient oscillation and eddy current contributions with bSSFP readout. Special algorithms for centric bSSFP acquisition such as the pairing order [36] may stabilize ALADDIN signals further. Spatial blurring may be introduced with the usage of bSSFP readout. The previous simulation studies showed that the perfusion signal of bSSFP-based ALADDIN can be reduced from 100% to ~40% during the acquisition of 192 bSSFP phase-encoding lines [18]. However, the problem of blurring is not specific to the bSSFP readout. The readout duration of a single-shot EPI with 64×64 matrix and 10 μs sampling time is ~40 ms, a value similar to T_2_* of tissue at 3T, indicating signal reduction of 100% to 37% during the EPI readout. Furthermore, blurring is inevitable in the 3D segmented approaches, such as 3D GRASE or 3D multiecho (RARE) stack of spiral, which are recommended for ASL imaging [24]. The blurring of bSSFP can be reduced with the usage of smaller PE steps. In this study, we used the same bSSFP readout scheme for all of ALADDIN, pCASL, and conventional MT asymmetry, in order to avoid the variations due to differences in the readout sequence for the comparison study.

It should be noted that ALADDIN is a data acquisition strategy rather than a labeling scheme and thus the conventional labeling schemes may potentially be incorporated into ALADDIN to improve the sensitivity. Currently, pCASL is the labeling scheme suggested for ASL, however, the scheme still suffers from signal loss in the region of long arterial transit time and from signal void when there are susceptibility artifacts in the region of the labeling plane (e.g. dental works, surgical pins in carotid artery) [24]. These features emphasize the requirement of developing a new ASL technique that is immune to heterogeneity in arterial transit time and also not affected by susceptibility artifacts in the region of teeth or neck which is spatially far from the actual imaging region (e.g., brain). ALADDIN PW imaging is different from any other ASL techniques in that the labeling planes automatically track the imaging plane in close proximity (Fig 1B), and thus is potentially less affected by heterogeneity in arterial transit time or the susceptibility artifacts from outside the imaging regions. ALADDIN is still in an early stage compared to the other mature conventional techniques such as pCASL, but has potential to accommodate all the advantages of the conventional techniques, while maintaining its own advantages such as robustness to heterogeneity in arterial transit time and susceptibility artifacts in the location of common labeling plane. Therefore, it is essential to investigate many different imaging sequences (which also work as the labeling sequences) such as fast low angle shot (FLASH) for ALADDIN in the viewpoint of the pCASL theory [8, 9], which warrants further investigation.

Because of superposition of signals from multiple labeling planes and unknown off-resonance distributions of flow spins, however, quantitative perfusion measurement with ALADDIN may be less accurate than the conventional ASL methods (Fig 4C). Several concerns exist in this regards. First, the blood labeling efficiency may be affected by many different imaging parameters such as TR, flip angle, slice thickness, gap, etc, which may be a limitation of using ALADDIN for quantitative measurements. Second, the transverse magnetization of the labeled blood spins may also contribute to the perfusion signals in the imaging slice, although assumed negligible in this study due to blood T_2_ (<300 ms) shorter than the time required for the labeled blood spins to move into the imaging region. Third, averaging contributions from different blood velocities may also contribute to inaccuracy in ALADDIN CBF quantification, although our simulation study showed that blood labeling efficiency varied little with blood velocity in ALADDIN (Fig 3A) (normalized standard deviation to be < 20%). Lastly, there is possibility that venous signals as well as arterial signals are contained within a single voxel in ALADDIN, where ALADDIN perfusion signals may decrease. Further studies are necessary to correctly address these problems to improve the accuracy of CBF quantifications with ALADDIN PW imaging.

Large inter-slice gap (e.g. 140%) used in this study can potentially become problematic for applying motion correction algorithms. Thus, investigations on the relationship between inter-slice gap and its effect on motion correction algorithms could be meaningful for future improvements of ALADDIN. This issue could be potentially resolved with usage of conventional gap by improving RF excitation profile. However, the smaller gap may make the perfusion signals affected by the cerebrospinal fluid, and thus should be used with cautions, as mentioned above.

ALADDIN is dependent on many imaging parameters and has potential for improvement through optimization. ALADDIN perfusion labeling is dependent on TR, matrix size, flip angle, phase-oversampling, and dummy PE steps, and thus the related parameters must be considered carefully to achieve desired perfusion labeling [18]. There is a possibility of improving the blood labeling efficiency of ALADDIN PW imaging by using an imaging sequence different from bSSFP, as mentioned above. The frequency offset for ALADDIN MTA imaging is dependent on the RF pulse bandwidth and the inter-slice gap [19], and thus adjusting the parameters may allow us to advance ALADDIN MTA imaging for sensitizing MT in free mobile peptides such as amide proton transfer (~3.5 ppm offset from the water resonance frequency) for multi-slice cellular pH imaging. The current ALADDIN MTA study focused on the effects of flip angle (i.e., saturation power), but not on TR and RF bandwidth. Preliminary studies showed that MTA has higher specificity to WM than MTR [37] and suggested that TR and RF bandwidth affect the MTA contrast which may be potentially related to myelination [38], all of which requires further investigation. These technical advancements can reduce the scan time and/or improve the image quality further. Furthermore, ALADDIN can potentially be sensitized to directionality of local blood flow in each pixel [39], map myelination [37], and quantitatively map MT signals [40]. These factors along with potential clinical usefulness of imaging both perfusion and MT-related information together warrant further investigation.

## Conclusion

ALADDIN is an interslice blood flow and MT imaging technique that potentially enables us to overcome the limitation of the conventional methods. The current study demonstrated that the blood flow, MT asymmetry, and MT ratio can be acquired simultaneously using the ALADDIN interslice blood flow and MT technique. ALADDIN perfusion and MT asymmetry signals showed similar sensitivity compared to the conventional methods emulating the first, second, and third prior slices of ALADDIN, suggesting ALADDIN signal to be superposition of signals from multiple labeling planes. The experimental results were in agreement with the simulation results. Along with the simultaneous imaging nature, the technique has great potential for improving the signal sensitivity and decreasing scan time further for better clinical utility, and thus further studies are necessary in this regard.
